# How to Cultivate the Sense of Smell
*"Olfactics and the Physical Senses.' By Charles Henry Piesse Member of the Royal College of Surgeons, Fellow of the Institute of Chemistry, Public Analyst, &c. (Published by Piesse and Lubin, 2, New Bond Street. 1887.)


**Published:** 1888-11-17

**Authors:** 


### How to Cultivate the Sense of Smell.*

(By a Consulting Physician.)

Use and practice bring the muscles of the limbs into a state of perfection for performing the functions to which they are put. On the other hand, neglect to use the muscles causes them to become weak, flabby, and more or less useless. What is true of the muscles is equally applicable to the mind, and to the five senses which keep guard over the body. The object of Mr. Piesse's work is to show the importance of cultivating the senses, and in particular the sense of smell; for, "this is the sense which stands guard over the air we breathe, and, to a great extent, over the food we take." But the sense of smell varies much in different people, some being almost entirely without it, while in others it is very acute. Mr. Piesse considers that persons who are unconscious of the existence of bad smells are so principally from neglect. "The sense of smell given by the Creator, as a guard against disease, has been neglected till it is naturally dead." We should not, however, attribute deficiency of the sense of smell so much to neglect as to habituation to bad smells and consequent insensibility to their presence. Indeed, in another part of his book, Mr. Piesse admits this, for he says, "constant exposure to foul odours renders the senses less and less acute, until the extreme point is reached, and the stage of complete anosmia (or loss of smell) is attained." Even in this London of ours, there are numbers of persons living in localities rivalling in stenches the City of Cologne. If in the words of Tennyson they do not literally live

"In healthless dungeons over steaming sewers,
Fed with rank bread, that crawled upon the tongue,
And putrid water, every drop a worm,
Until they died of rotted limbs"

—if they do not literally live thus, they reside under such conditions as must necessarily blunt not only the sense of smell but all the senses, and both the moral and physical faculties. Bad odours have long been supposed to affect the moral faculties. Hamlet talks of "beasts from hell"; and in 1793 the peculiar wickedness of the people who lived in the neighbourhood of Etna and Vesuvius was asserted to be caused chiefly by the smell of sulphur, which is constantly discharged from those volcanoes. It is, however, quite possible that when people exposed to bad air do not lose the sense of smell they may grow to prefer so-called bad smells to the scent of flowers. For there are those to whom the latter is disagreeable, while the scent of high game or of sulphur is pleasant to them. Mr. Piesse refers this to peculiar idiosyncracy. Mr. Piesse remarks, "Of all the ideas inculcated into the infant's mind by a mother's love and care, there is none so important as the sense of smell. A child's sense of refinement will greatly depend upon the extent to which this sense is cultivated or neglected." If this is the case, there can be small hope for the children of those living near "steaming sewers." In fact, only those living in the country can hope to impart such perfection to the education of their children. That the sense of smell can be educated to very great perfection is evident from the statement made that "an experienced perfumer may have 200 odours in his laboratory, and can distinguish every one by name." It is also stated that the sense of smell will recognise air containing the 200,000th part of bromine vapour, the 1,300,000th part of a grain of otto of roses, and the 13,000th of a grain of musk. The nautical novelist, Maryatt, seems to have been well acquainted with the great diffusibility of scents, for he tells how a man-of-war chased a flying vessel carrying otto of roses by the scent left behind! Other odours having great diffusibility are the Rosemary of the Spanish coast, which is perceptible long before land comes in sight, and the Plumeria of the West Indies. The odour of wet earth is another scent having great diffusibility. This is particularly the case in tropical countries, where, after a long period of dry weather, the rainy season commences, the odour of the wet earth being perceptible long before the rain reaches the locality. Then there is the "smell of blood," which, although not of great diffusibility, is popularly supposed to cling to a person or object for a lengthened period. This, however, is doubtless a delusion, or we might not have so much difficulty in capturing murderers!

There is no doubt that the sense of smell may often serve to protect us from the danger of disease. The amount of disease caused by drinking impure water is considerable, and the causes of water becoming impure are numerous. A dead rat or a dead mouse in the tank, black beetles in the kitchen boiler, neglected dustbins, damp, mildew, defective gas fittings, dirty linen, are all to be detected by the sense of smell, to say nothing of more powerful odours such as emanate from drains, sewers, middens, and foul privies. The cause of odours is generally admitted to be minute particles which evaporate from the odorous substance coming into contact with the olfactory organ. "And," asks Mr. Piesse, "what safeguard have we against inhaling such infinitesimally small particles except a highly-cultivated nose? But while the eye and the ear have teachers the education of the nose is left to chance. A foxhound, a pointer, or a terrier may be trained to a more quick or precise scent, but to speak of educating our own noses only provokes a smile. We learn of a person possessing a marvellous ear for music, of another being a profound connoisseur of art, of a third possessing a perfect delicacy of touch, but should some unfortunate have his other two senses —smell and taste—equally well-educated, he runs the risk of being rated a dainty fop. Some people are, however, of opinion that it must be rather a drawback, having an acute sense of smell. There is a popular idea that it renders people liable to cold, and to catch contagious disorders, or to hay fever. Mr. Piesse says, nothing at all of the kind. An acute sense of smell renders the person capable of enjoying agreeable odours, which, as a rule, are harmless, and of detecting disagreeable odours, which may have a bad effect. There is the army of disease stalking amongst us led by the Demon Pestilence, which has done far more to destroy and depopulate than all the battles ever fought. "The van-guard of this army is invariably 'stink.' We use the coarse word rather than attempt to mince matters. The only way of getting rid of stinks is to teach people to shun them" by the education of their noses. "To a man with an educated nose death would be preferable under a sweet-smelling haystack to life under the conditions that some seem to bear unconsciously."

We cannot, however, help thinking that there is just a little hobby-riding in Mr. Piesse's book. If all from infancy were placed in a position so that no impressions from bad smells should be made, the education of the nose up to the pitch desired by the author could no more be accomplished than education in music or in painting could be universally accomplished. Some are naturally more endowed with acute perception in one direction, and some in another. Perhaps, in the course of generations, a people might be produced, all having acute sense of smell. But under our conditions of life the idea is chimerical. We should have to return to the primitive condition of the human races, among whom smell was very acute, as among savages of the present day. Again, sulphur odour is stigmatised. The poetical idea of hell is "sulphurous and tormenting flames," and it has been already mentioned that peculiar wickedness has been attributed to sulphurous air. Sulphur odour is to be shunned by the educated nose. But sulphur is one of the principal disinfectants we have, and is constantly in use as such. Again it is by no means certain that as a rule offensive odours have a bad effect. Odours and causes of disease are not inseparable, they may or may not be combined. But as they may be combined, it is of course better to shun bad odours. We cannot quite accompany our author when he jumps the gate (as previously quoted), that nothing is so important to be inculcated into the infant's mind as the sense of smell; important it may be, but the expression is just a little too strong.

There are many other interesting subjects touched upon in Mr. Piesse's book. For instance, he goes into the debated question whether vultures and condors are guided by the

* "Olfactics and the Physical Senses.' By Charles Henry Piesse Member of the Royal College of Surgeons, Fellow of the Institute of Chemistry, Public Analyst, &c. (Published by Piesse and Lubin, 2, New Bond Street. 1887.)

sense of sight or the sense of smell, the balance of evidence being that sight not smell guides birds of prey. There is also an interesting disquisition on the connexion between sound and odour, and a gamut of odours, forty-six in number, are placed side by side with their equivalent in musical notes. Odours are also coupled with recollection, rosemary being for remembrance. But ideas of all kinds are very closely linked together. We never smell chloride of lime without thinking of cholera, nor sage and onions without thinking of roast pig and Charles Lamb. Mr. Piesse also tells us of the antiquity of the use of odours, especially in the conduct of religious ceremonies. We have it in evidence that the Lord himself did not disdain to smell a sweet savour. Incense in churches has been used from the earliest periods. The Greeks and Romans were especially fond of scents, especially in their baths, and in the East in the present day many perfumes are in much greater use than among Western nations. Perfumes also have been much used in physic. In "Cotgraves Treasury of Wit," 1655, we have an announcement by a professor of the healing art who recites:

"My name is Pulsefeel, a poor doctor of physic,
I can clarifie your blood, supple your cheeks, perfume your skin, tinct your hair, enliven your eye."

There is also some information with regard to the manufacture of perfumes. The purchase of the raw material for perfumery depends on the nose. There is a popular impression that various perfumes are distilled from very nasty material. Mr. Piesse does not however tell us this. Perfumes are made by *enfleurage*, or enflowering, and not from unpleasing objects, neither does Mr. Piesse tell us "where the flower hides her scent, or from what cup of hidden sweets she sucks it."

Before concluding our remarks on this very interesting book, we may mention that we are glad to find Mr. Piesse cautioning against the practice of overpowering a bad odour by the presence of a pleasant one. This is not disinfection, and it is worse than useless. Deodorants are not usually disinfectants, and to overcome disease we require the latter. Mr. Piesse advocates the use of the "Ribbon of Bruges," in the sick chamber, as being both a deodorant and a disinfectant.

